# Admission blood tests predicting survival of SARS-CoV-2 infected patients: a practical implementation of graph convolution network in imbalance dataset

**DOI:** 10.1186/s12879-024-09699-x

**Published:** 2024-08-09

**Authors:** Jie Lian, Fan Huang, Xinhai Huang, Kitty Yu-Yeung Lau, Kei Shing Ng, Carlin Chun Fai Chu, Simon Ching Lam, Mohamad Koohli-Moghadam, Varut Vardhanabhuti

**Affiliations:** 1 Department of Diagnostic Radiology, Li Ka Shing Faculty of Medicine, The University of Hong Kong, Pokfulam, Hong Kong SAR China; 2 Faculty of Science, The University of Hong Kong, Pokfulam, Hong Kong SAR China; 3 WHO Collaborating Centre for Infectious Disease Epidemiology and Control, School of Public Health, Li Ka Shing Faculty of Medicine, The University of Hong Kong, Pokfulam, Hong Kong SAR China; 4 Department of Computing, The Hang Seng University of Hong Kong, Shatin, Hong Kong SAR China; 5 School of Nursing, Tung Wah College, Ho Man Tin, Hong Kong SAR China

**Keywords:** COVID-19, Graph convolutional networks, Machine learning, Cox Proportional-Hazards, Survival prediction

## Abstract

**Background:**

Predicting an individual’s risk of death from COVID-19 is essential for planning and optimising resources. However, since the real-world mortality rate is relatively low, particularly in places like Hong Kong, this makes building an accurate prediction model difficult due to the imbalanced nature of the dataset. This study introduces an innovative application of graph convolutional networks (GCNs) to predict COVID-19 patient survival using a highly imbalanced dataset. Unlike traditional models, GCNs leverage structural relationships within the data, enhancing predictive accuracy and robustness. By integrating demographic and laboratory data into a GCN framework, our approach addresses class imbalance and demonstrates significant improvements in prediction accuracy.

**Methods:**

The cohort included all consecutive positive COVID-19 patients fulfilling study criteria admitted to 42 public hospitals in Hong Kong between January 23 and December 31, 2020 (*n* = 7,606). We proposed the population-based graph convolutional neural network (GCN) model which took blood test results, age and sex as inputs to predict the survival outcomes. Furthermore, we compared our proposed model to the Cox Proportional Hazard (CPH) model, conventional machine learning models, and oversampling machine learning models. Additionally, a subgroup analysis was performed on the test set in order to acquire a deeper understanding of the relationship between each patient node and its neighbours, revealing possible underlying causes of the inaccurate predictions.

**Results:**

The GCN model was the top-performing model, with an AUC of 0.944, considerably outperforming all other models (*p* < 0.05), including the oversampled CPH model (0.708), linear regression (0.877), Linear Discriminant Analysis (0.860), K-nearest neighbours (0.834), Gaussian predictor (0.745) and support vector machine (0.847). With Kaplan-Meier estimates, the GCN model demonstrated good discriminability between low- and high-risk individuals (*p* < 0.0001). Based on subanalysis using the weighted-in score, although the GCN model was able to discriminate well between different predicted groups, the separation was inadequate between false negative (FN) and true negative (TN) groups.

**Conclusion:**

The GCN model considerably outperformed all other machine learning methods and baseline CPH models. Thus, when applied to this imbalanced COVID survival dataset, adopting a population graph representation may be an approach to achieving good prediction.

**Supplementary Information:**

The online version contains supplementary material available at 10.1186/s12879-024-09699-x.

## Background

Since the emergence of SARS-CoV-2 in 2019, it has continued to impact individual health systems. One important cornerstone in the management is to identify those at risk of mortality, and judicious hospital admission for those at higher risk so as to appropriately direct hospitalisation for the most needed. This is particularly important in phases where there is a high number of infections so as to not overwhelm the health systems. There is an ever-increasing body of research focusing on various areas ranging from detection, diagnosis, and prognosis to survival prediction, which not only involves traditional clinical assessment, but also increasingly artificial intelligence that has been applied with promising results.

Previous attempts with machine learning algorithms to investigate clinical data sets of COVID-19 patients with known results include the use of decision trees, random forests, variants of gradient boosting machines, support vector machines, and K-nearest neighbours and deep learning methods [1–3]. Early approaches have focused on the so-called enriched datasets, whereby the outcome in question (e.g. mortality) is increased in proportion so that they are matched in the training datasets. This approach has generally produced good predictive results for mortality prediction. However, the real-life incidence of mortality, for example, is low relative to the whole population, and in real-life application owing to the severely imbalanced datasets, this will usually result in unsatisfactory predictive performance. More specifically, the trained machine learning model will always classify the minority category into the majority category because of a lack of learning information from the minority samples. Although the accuracy values may be inflationary high, the predictor’s performance is still unsatisfactory in many aspects. We have to consider not only the accuracy but also the sensitivity, specificity of each category, as well as precision and F_beta_ score [4, 5]. To this end, several methods have been proposed to deal with the imbalanced dataset such as synthetic minority oversampling technique (SMOTE) [6]. The ability of machine learning and deep learning to satisfactory deal with imbalanced datasets remains an open problem.

Comparing with conventional clinical analysis model and traditional machine learning methods, graph convolutional neural network (GCN) can be considered as an inductive framework which helps generalising the model to handle unseen data more effectively [7, 8]. It provides a set of aggregating functions to consolidate information from a node’s local neighbourhood, which in turn enables the model to exploit the structural relations among multiple data types more effectively despite the limited data sample. As a result, the use of GCN may alleviate the undesirable influences caused by the imbalanced dataset in this study. In recent years, graph presentation has been increasingly popular in the medical community being applied at a patient level, and mostly at an organ level connection [9–11]. Some examples include the utilisation of graph theory in the interconnection of neurons in the brain [10], and the vascular connections of retinal vessels [11]. Inspired by the notion of communication network, we can also build a graph network instead of applying at a patient level, but from a population level [12].

The purpose of this study is to predict the survival of SARS-CoV-2 infected patients using admission blood tests and a population-based graph convolutional network (GCN) model. As a proof of concept, we apply simple laboratory blood tests to predict patient survival and compare the GCN model’s performance with traditional machine learning and oversampling methods. We hypothesize that the GCN model will outperform traditional ML methods in handling severely imbalanced datasets.

## Methods

The study design followed the TRIPOD protocols for Prediction Model Development and Validation (see Supplementary I).

### Study design and participants

The Hong Kong Hospital Authority Clinical Data Analysis and Reporting System (CDARS) was used to search patients’ electronic data consisting of 42 public hospitals in Hong Kong’s territory. Patients with a positive test based on a reverse-transcriptase polymerase chain reaction (RT-PCR) test for SARS-CoV-2 that met the testing standards provided by the Centre for Health Protection, Department of Health, and Government of Hong Kong SAR were included. The cohort was retrieved for all consecutive patients from the first positive patient admission in Hong Kong between January 23 to December 31, 2020.

Observational data, comprising demographics (age and gender) and eighteen basic laboratory blood tests (white blood cell count (WBC), neutrophil count (NEUT), lymphocyte count (LYM), monocyte count (MON), haemoglobin (HGB), haematocrit (HCT), platelet (PLT), albumin (Alb), total bilirubin (TBIL), alanine aminotransferase (ALT), alkaline phosphatase (ALP), lactate dehydrogenase (LDH), creatine kinase (CK), urea, creatinine (Cr), C-reactive protein (CRP), sodium (Na) and potassium (K)) were retrieved on admission day.

#### Attribute density analysis

To understand the distribution and completeness of our dataset, we conducted an attribute density analysis. This analysis included plotting the distribution of each attribute for the normal and deceased cohorts, segmented by overall population, male, and female groups. Attributes with a high percentage of missing values were imputed using multiple imputation methods. Detailed distributions are provided in Supplementary Fig. 1.

**Fig. 1:**
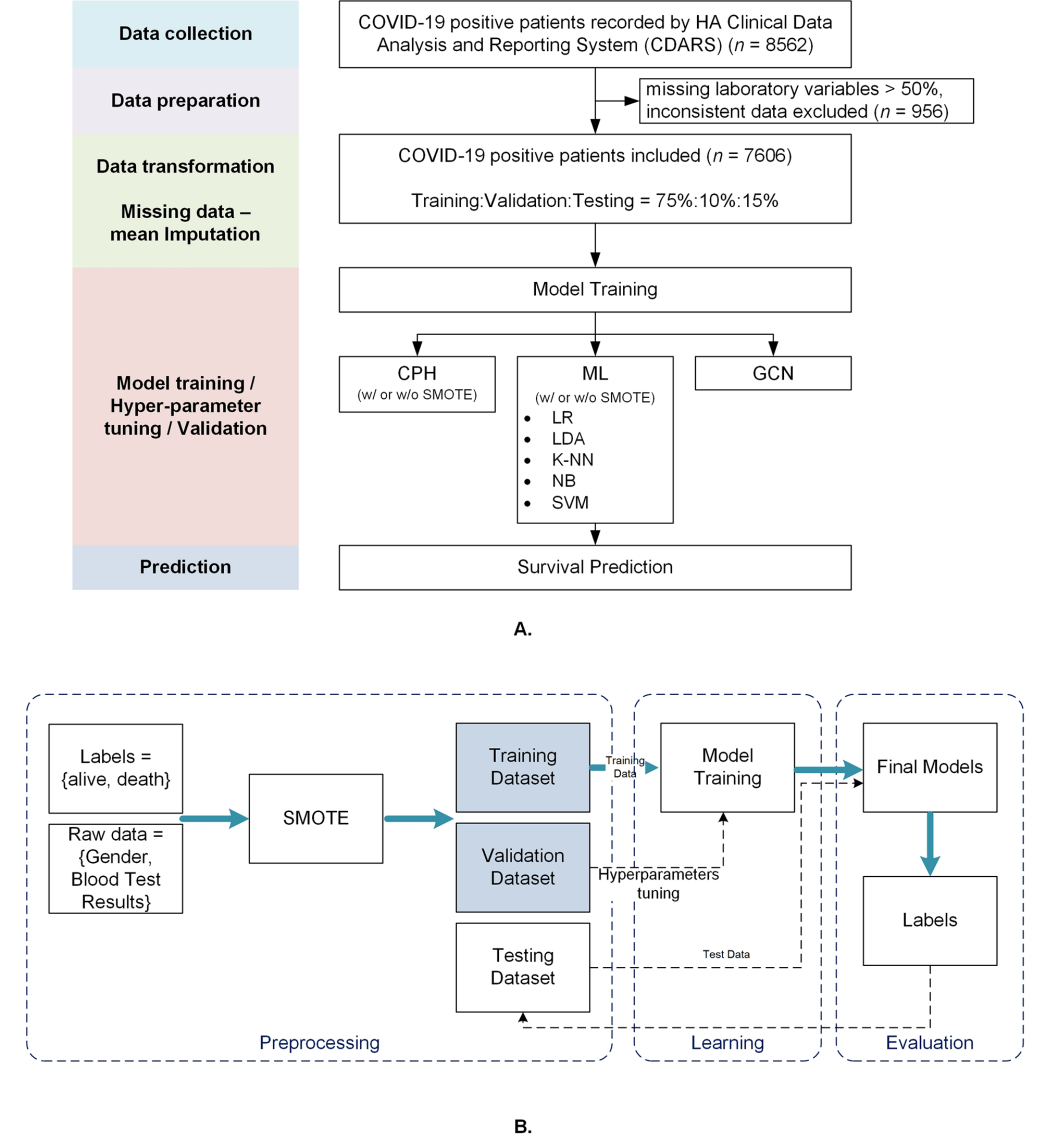
Overview of the pipeline used for survival prediction and oversampling. **(A)** Flow Chart of the Study Design and Participants. **(B)** Traditional ML model with the SMOTE techniques. The training and validation set were applied with SMOTE, followed by model training and tuning. The testing set was processed without SMOTE

Cases with missing blood tests with less than half of the available data were excluded. Cases remaining with missing data less than 50% were imputed by the mean of non-missing cases of that variable. In addition, data on mortality were obtained for each patient 45 days after discharge and included those deemed related to COVID-19 based on the recorded cause of death information. For details of data preparation, please refer to Fig. 1(A) and supplementary II.

Institutional review boards approved this study protocol in multiple hospitals across Hong Kong HKU/Hong Kong West Cluster Research Ethics Committee (Ref. UW 20–291), Hong Kong East Cluster Research Ethics Committee (HKECREC-2020-012), Kowloon Central/Kowloon East Cluster Research Ethics Committee (KC/KE-20-0052/ER-3), Kowloon West Cluster Research Ethics Committee (Ref. KW/EX-20-065), CUHK/New Territories East Cluster Clinical Research Ethics Committee (Ref. 2020.216), and New Territories West Cluster Research Ethics Committee (NTWC/REC/20048). Due to the retrospective nature of the study, the various ethics committee approved data usage and informed consent was waived for the patients used in this study.

### Experimental design

Three sets of experiments were designed separately to evaluate the effectiveness of the mortality prediction at admission in (1) traditional machine learning models, (2) oversampling machine learning models, (3) GCN model prediction performance on the unbalanced dataset. Specifically, the entire HK population blood test dataset was randomly stratified and separated into three portions: 75% for training, 10% for validation, and 15% for testing. We also set the Cox proportional-hazards (CPH) models as our baseline model. In addition, we applied oversampling method on dataset and trained a CPH based on the oversampled dataset.

### Traditional machine learning model development

We utilised five most commonly used machine learning models (see Supplementary 111) to predict the mortality, namely Logistic Regression (LR) [13], Linear Discriminant Analysis (LDA) [14], K-Nearest Neighbours (KNN) [15], Gaussian process [16], Support Vector Machine (SVM) [17], and XGBoost. All models were trained on the training and validation sets, while the validation set is a set of data separated during model training that was used to adjust the model’s hyper-parameters (e.g. the choice of penalty function), and finally tested on the separate held-out test set. Besides, on order to suggest our model’s generality, we also added a set of five-folder cross validation experiments. shou Due to the high imbalance of the positive and negative cases in our dataset, which as prior discussed may give rise to the poor learning ability of the model, we further used the SMOTE to create a more balanced data set for the training and validation data. This kind of oversampling method was regarded as a common technique used to improve the performance of the machine learning model in many fields [6, 18, 19]. We also compared SMOTE with random oversampling and underdsamping methods. The whole oversampling training process is shown in Fig. 1(B).

### Population graph construction and model construction

**Fig. 2:**
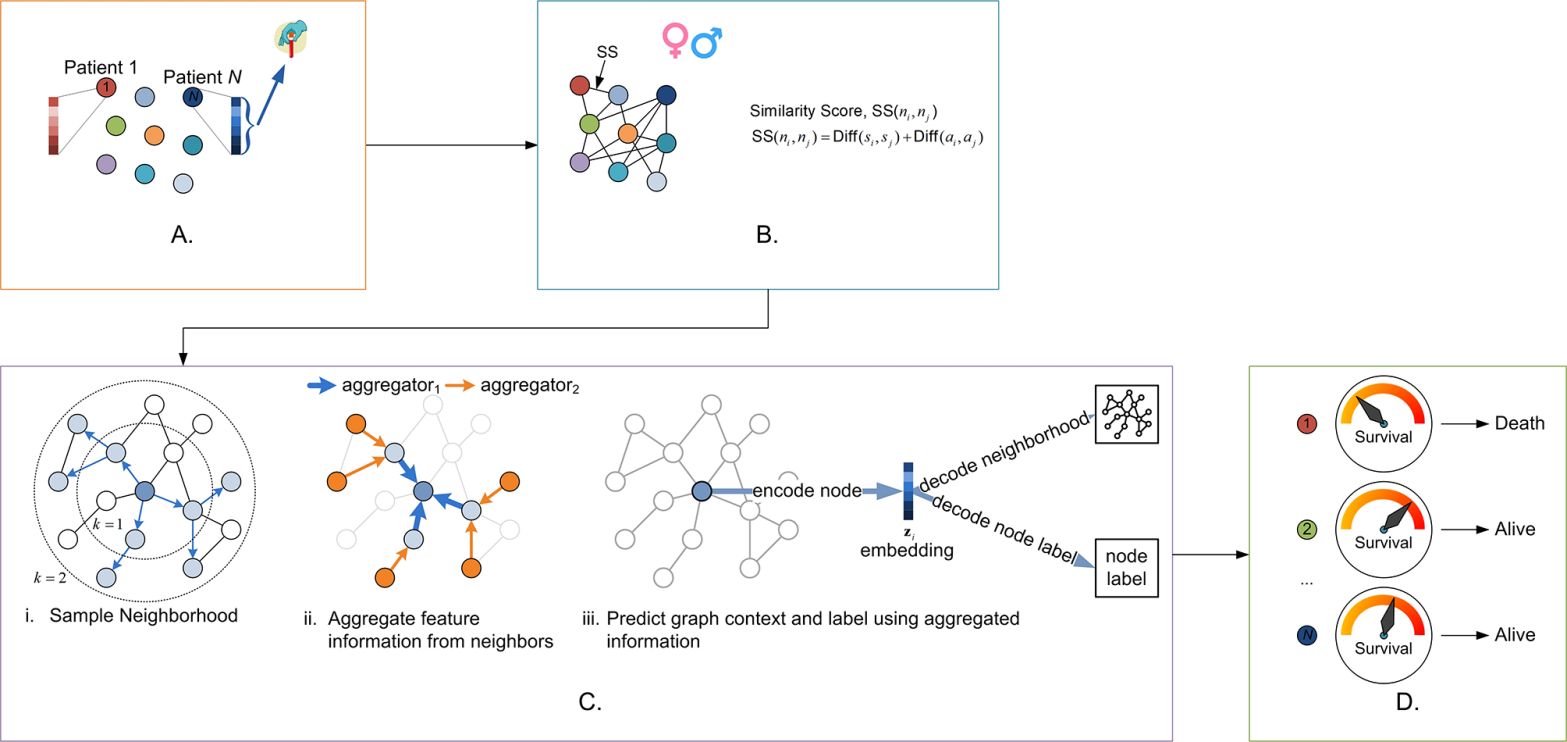
Overview of the pipeline used for survival prediction of population graphs using Graph Convolutional Networks. **(A)** Patients were used as nodes. Blood test values were used as nodes feature vectors. **(B)** Population-based graph was based on similarity scores based on age and gender. **(C)** Incorporation of the population graph into a two-layer GraphSAGE network. **(D)** The final step of survival predictions

In this project, we applied a graph representation to model the population COVID-19 survival data. When designing a general graph model, two important elements needed to be considered. First, the choices of nodes along with their features, and second the edges which describe the nodes’ interactions. In this study, we considered the laboratory blood test data and non-laboratory data (age and sex) as important elements to build a population-based graph. Specifically, we defined each single patient as a node sample, along with blood test values as the node feature vector in our model (see Fig. 2(A)). The interactions between each pair of patient’s nodes were described as edges in the graph, which was based on the patients’ age and sex similarity (see Supplementary IV for the detailed explanation). In this case, if two patients share a similar age range and were of the same gender, their similarity score will reach the maximum value, thereby being represented as very close neighbours in a graph representation (see Fig. 2(B)).

Graph convolution neural network is aimed at learning a graph embedding state based on the neighbourhood information for each node. Among all graph neural network structures, an inductive framework called SageConv [20] which permits updating node features by sampling and aggregating information from the node’s local neighbourhood, has shown strong performance on large graphs. We use a two-layer SageConv structure with a sum-readout layer as our survival prediction model in this paper. The resultant algorithm will then generate a survival label for each patient. Once the population-based graph has been built, it is regarded as the input of the GCN network and every node’s feature vector was updated based on their neighbour’s status during the training process, converging to a stable state in the end to be used as final survival features (see Fig. 2(C-D)).

### Model implementation

In the first experiment, a set of traditional ML models was trained on the training set using the scikit-learn python library (version1.0.1.) [21], followed by hyper-parameter selection on the validation set. The oversampling machine learning models were then developed using the same Python package as the traditional one, with the training and validation datasets subjected to a SMOTE technique prior to training the models. For the graph convolutional neural network model, the Deep Graph Library (version 0.6.1.) [22] was used via Python implementation. When training, the pre-constructed population graph was regarded as the input for our graph neural network, which consisted of two graph-convolution units, a SageConv graph layer, a ReLU activation layer, and a dropout layer separately, followed by a fully connected layer for prediction. The training epoch number was set to 1000 while an early-stopping function and reduced-LR method were added to avoid overfitting and find an optimal learning rate, along with a weighted loss computed from the distribution of training labels and a dropout value of 0.2.

### Population graph sub-analysis

When a well-performing model was obtained, the next step was to understand why the model made such predictions. As the entire population was embedded as a graph, some graph properties can be adapted to conduct further analysis. A graph-structured analysis metric based on weighted-in score was defined and calculated on the testing samples after the survival prediction task. We proposed a weighted-in function that calculated the sum value for each patient node on the graph by adding the weights of all edges that connect to the node, i.e., for patient *i* with node *n*_*i*_, the weighted-in score of *n*_*i*_ is $w{i_{{n_i}}} = \sum {_s{w_{{n_s}}}} \,\,\,\,\,w{i_{{n_i}}} = \sum {_s{w_{{n_s}}}}$, for any node *n*_*s*_ connected with *n*_*i*_. The weighted-in score can be thought of as a weighted connectivity property, and it only focuses on the graph structure information, allowing for visualisation of how powerful graph representation is. Thus, we further implemented a graph structure analysis based on the weighted-in score in the testing subgroups using Python’s Seaborn package (version 0.11.2) [23]. All experiments were implemented in Google Colab platform with a default hardware setting.

### Statistical analysis

Independent sample t-test values were calculated to compare the means of different groups. These values help determine if there are statistically significant differences between the survival outcomes of different patient cohorts. A *p*-value of less than 0.05 was considered statistically significant. True-positive (TP), false-positive (FP), true-negative (TN), and false-negative (FN) outcomes were calculated based on confusion matrices. The estimated specificity threshold was set at 0.5. To assess the performance of a model, areas under the receiver operating characteristic curve (AUC), Accuracy, sensitivity (recall), specificity, positive predictive value (precision), and F_**10**_ score [4, 5] were calculated. We applied F_**10**_ instead of F_**1**_ to emphasize the models’ ability to detect true positive cases in this extremely imbalanced dataset. DeLong test [24, 25] was used to compare AUC of different models. Comparison between the weighted-in cohorts was performed using Wilcoxon test. A *p*-value of < 0.05 was deemed as statistical significance. We then performed a survival analysis using Kaplan-Meier estimates for low- and high-risk patients, as well as a log-rank test, using the scores predicted by the baseline, best performed ML and GCN models on the testing set. A Cox proportional-hazard model was used to calculate the hazard ratio of our GCN biomarker.

## Results

Our dataset included a total of 7606 COVID-19 confirmed patients, including 142 deceased patients (see Table 1). The majority of patients were female (*n* = 3909, 51.4%) with mean age (46.94 years old, 95% CI (46.51–47.37)). Independent sample t-test values indicated that the blood parameters were significantly different between the survived and deceased cohorts. Specifically, the t-test values showed statistically significant differences (*p* < 0.05) in most parameters, underscoring the relevance of these biomarkers in predicting survival outcomes. When sub-analysis based on gender was performed, all blood parameters except for male patients of TBIL (*p* = 0.244) and ALT (*p* = 0.882) were statistically significant. (See Supplementary II).

**Table 1:** Demographics and clinical characteristics of 7606 COVID-19 positive patients

		All patients, *n* = 7606	Deceased cohort, *n* = 142
Full Name	(Unit; Normal reference range)	Mean ± SD (95% CI)	
Age	Years	46·94 ± 19·15, (46·51 − 47·37)	79·41 ± 11·04, (77·59–81·22)
Survival days after onset	Days	NA	17·93 ± 16·29, (15·23 − 20·63)
Gender (Male)	Count; %	3697 (48·6%)	87 (61·27%)
Haemoglobin	(g/dL; 11·7–14·9)	13·56 ± 1·65, (13·53 − 13·60)	12·02 ± 2·11, (11·68 − 12·37)
Haematocrit	(L/L; 0·35 − 0·45)	0·40 ± 0·05, (0·40 − 0·40)	0·36 ± 0·06, (0·35 − 0·37)
White Blood Cell count	(10^9^/L; 3·7–9·2)	5·66 ± 2·14, (5·61 − 5·71)	7·81 ± 3·88, (7·17 − 8·45)
Neutrophil count	(10^9^/L; 1·7 − 5·8)	3·57 ± 1·85, (3·53 − 3·62)	5·98 ± 3·55, (5·40 − 6·56)
Monocyte count	(10^9^/L; 0·1 − 0·8)	0·54 ± 0·24, (0·54 − 0·55)	0·64 ± 0·43, (0·57 − 0·71)
Lymphocyte count	(10^9^/L; 1·0–3·1)	1·44 ± 0·79, (1·42 − 1·46)	1·07 ± 0·67, (0·96 − 1·18)
Platelet	(10^9^/L; 145–370)	224·58 ± 75·89, (222·88–226·29)	195·55 ± 77·33, (182·83–208·27)
Sodium	(mmol/L; 136–145)	138·11 ± 3·12, (138·04–138·18)	136·72 ± 4·94, (135·91–137·53)
Potassium	(mmol/L; 3·4–4·8)	3·81 ± 0·44, (3·80 − 3·82)	4·03 ± 0·62, (3·93 − 4·14)
Creatinine	(µmol/L; 49·0–90·0)	74·96 ± 46·00, (73·93 − 75·99)	128·75 ± 104·24, (111·60–145·89)
Urea	(mmol/L; 2·8–8·1)	4·31 ± 2·30, (4·26 − 4·36)	8·84 ± 6·30, (7·81 − 9·88)
Albumin	(g/L; 35·0–52·0)	40·12 ± 4·99, (40·01–40·23)	33·57 ± 6·23, (32·55 − 34·60)
Alkaline phosphatase	(µ/L; 30–120)	74·78 ± 43·17, (73·81 − 75·75)	92·14 ± 85·77, (78·03 -106·24)
Total bilirubin	(µmol/L; 5·0–21·0)	8·92 ± 5·44, (8·80 − 9·05)	10·53 ± 13·22, (8·36 − 12·71)
Alanine aminotransferase	(µ/L; 0·0–34·4)	31·81 ± 49·41, (30·70 − 32·92)	61·02 ± 295·77, (12·37–109·67)
Lactate dehydrogenase	(µ/L; 0·0-246·4)	213·64 ± 82·97, (211·78–215·51)	310·45 ± 177·17, (281·31–339·59)
Creatine kinase	(µ/L; 39–308)	144·98 ± 285·39, (138·56–151·39)	270·57 ± 569·29, (176·94–364·21)
C-reactive protein	(mg/dL; 0·0–5·0)	1·64 ± 3·36, (1·57 − 1·72)	6·30 ± 7·07, (5·13 − 7·46)

The original dataset (7606) was divided by simple random sampling approach into training dataset (*n* = 5704, 75%), validation dataset (*n* = 761, 10%) and testing datasets (*n* = 1141, 15%). The oversampled version from the original dataset contains a training dataset (*n* = 11204), and a validation set (*n* = 1492). Independent sample t-test shows the training/validation data set are comparable to the testing data set. (see Supplementary Table 1.)

We first compare the SMOTE method with random over-and under sampling methods (Supplementary Fig. 2) using five-folder cross validation. SMOTE shows much improvement compared with random undersampling on all models, while slight improvement compared with random oversampling.

**Fig. 3:**
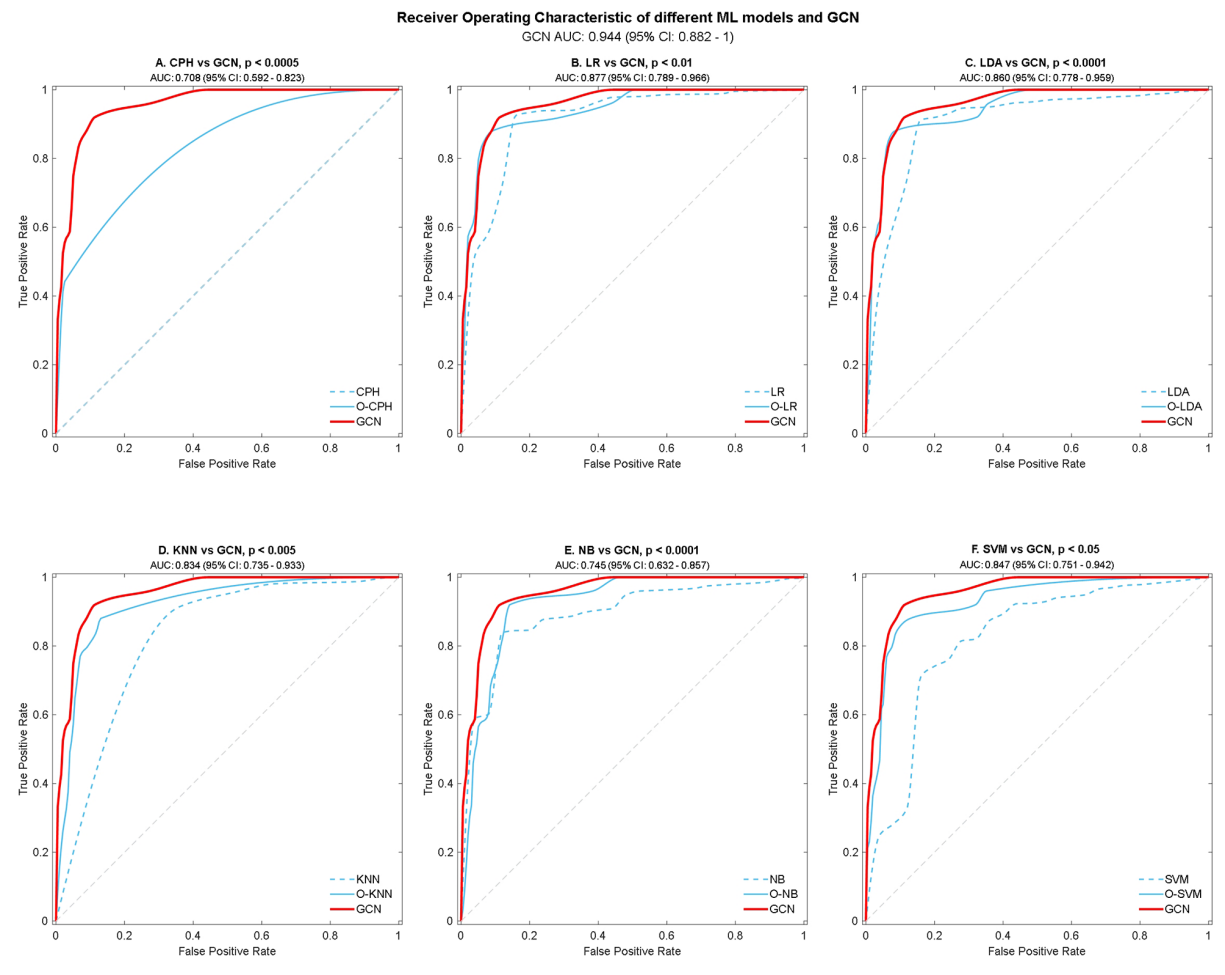
Performance of CPH models and ML models, comparing with GCN, with 95% CI, is given for each parameter. The diagonal dividing the ROC space represents the random event

We then report the baseline models, the non-oversampling CPH model received poor performance with the AUC of 0.49, while increased to 0.708 after oversampling (Fig. 3(A)). We then compared the traditional ML models, oversample traditional ML models, and the GCN models without oversampling separately (please refer to Table 2; Fig. 3). Among all traditional ML models, the Gaussian predictor performed the best, with an AUC of 0.736 and an accuracy of 94.2%, followed by the LDA model, which achieved an AUC of 0.610 and an accuracy of 96.3%. Regarding the other models, while they all achieved a good level of accuracy, their AUC values ranged from (0.50 to 0.74), which was expected given the very imbalanced nature of the dataset for training and testing. The best performing oversampling ML was the LR (O-LR) model achieved the best performance, with an AUC of 0.877 and an accuracy of 91.3%, respectively, compared to 0.500 and 97.8% using the traditional methods. Additionally, the AUC values for the oversampled LDA (O-LDA) and SVM (O-SVM) models were increased to 0.869 and 0.847, respectively, compared with 0.610 and 0.500 before. The O-XGBoost model reached an AUC of 0.725 and an accuracy of 95.9% after SMOTE, compared to 0.559 and 97.9% The GCN model was the best performing model out of all the techniques, with an AUC of 0.944 and an accuracy of 0.909 on the testing dataset after 200 epochs(please refer to Supplementary Table 2 for hyper-parameter list). The AUC of GCN significantly outperforms other ML models’ performances based on DeLong’s test (*p* < 0.05) (please refer to Supplementary Table 3). Moreover, the five-folder cross-validation experiments (Supplementary Table 4) also suggested that our GCN model has the highest mean of AUC score comparing with all the oversampling ML models.

**Table 2:** Performance of different machine-learning algorithm with GCN models on testing dataset

	ML models	AUC (95% CI)	Accuracy (95% CI)	Sensitivity (95% CI)	Specificity (95% CI)	Precision (95% CI)	F_10_ Score (95% CI)
**Traditional Models**	**LR**	0·5 (0·386–0·614)	0·978 (0·970–0·987)	0 (0–0·133)	1 (0·997–1)	0.428 (0.158–0.749)	0.121 (0·109–0·134)
	**LDA**	0·610 (0·491–0·728)	0·963 (0·952–0·974)	0·240 (0·115–0·434)	0·979 (0·969–0·986)	0·207 (0·059 − 0·354)	0·240 (0·223–0·256)
	**KNN**	0·5 (0·386–0·614)	0·978 (0·970–0·987)	0 (0–0·133)	1 (0·997–1)	NaN **	0 (0–0·001)
	**GAUSSIAN**	0·736 (0·623–0·849)	0·942 (0·929–0·956)	0·520 (0·335–0·700)	0·952 (0·937–0·963)	0·194 (0·099 − 0·289)	0.511 (0.492–0·531)
	**SVM**	0·450 (0·385–0·614)	0·977 (0·969–0·986)	0 (0–0·133)	0·999 (0·995–1)	0 (0–0)	0 (0–0·001)
	**XGBoost**	0.559 (0.441–0.677)	0.979 (0.971–0.987)	0.120 (0.042–0.300)	0.998 (0.993–0.999)	0.600 (0.231–0.882)	0.121 (0.109–0.134)
**Traditional Models based on oversampled data**	**O-LR**	0·877 (0·789–0·966)	0·913 (0·897–0·930)	0·840 (0·653–0·936)	0·915 (0·897–0·930)	0·181 (0·111 -0·251)	0·810 (0·794–0·824)
	**O-LDA**	0·860 (0·778–0·959)	0·858 (0·838–0·878)	0·880 (0·700–0·958)	0·863 (0·841–0·882)	0·126 (0·077 − 0·175)	0·830 (0·815–0·843)
	**O-KNN**	0·834 (0·735–0·933)	0·904 (0·887–0·921)	0·760 (0·566–0·885)	0·908 (0·889–0·923)	0·156 (0·091 − 0·220)	0·545 (0·526–0·564)
	**O-GAUSSIAN**	0·745 (0·632–0·857)	0·921 (0·905–0·937)	0·560 (0·371–0·733)	0·930 (0·914–0·944)	0·152 (0·079 − 0·226)	0·732 (0·714–0·748)
	**O-SVM**	0·847 (0·751–0·942)	0·930 (0·915–0·945)	0·760 (0·566–0·885)	0·934 (0·918–0·947)	0·204 (0·122 -0·286)	0·739 (0·722–0·756)
	**O-XGB**	0.725 (0.610–0.838)	0.959 (0.947–0.970)	0.480 (0.300–0.665)	0.967 (0.957–0.978)	0.261 (0.156–0.403)	0.476 (0.457–0.495)
**Our proposed method**	**GCN**	0·944 (0·882–1)	0·909 (0·892–0·926)	0·880 (0·700–0·958)	0·909 (0·891–0·925)	0.179 (0·121–0.256)	0·847 (0·833–0·860)

*O-means oversampled

**TP = FP = 0

With the exception of the GAUSSIAN approach, practically all traditional machine learning models have a F_**10**_ score value of less than 0.3. After oversampling, the performance of all machine learning models was greatly enhanced, and the oversampled LDA obtained a maximum value of 0.830, up from 0.24 previously. Nonetheless, the GCN predictor had the highest F_**10**_ score (0.847) of any model.

As for the survival analysis, the median stratification of patient prediction scores in the training set to distinguish between low and high-risk groups. The oversampled CPH, oversampled LR and GCN model all showed a strong ability to separate low- and high-risk people with *p*-values smaller than 0.0001. The hazard ratio for GCN biomarker is 4.20 (CI: 2.99–5.20, *p* < 0.0001). The detailed KM curver can be found in Supplementary Fig. 4.

### Population graph sub-analysis

**Fig. 4:**
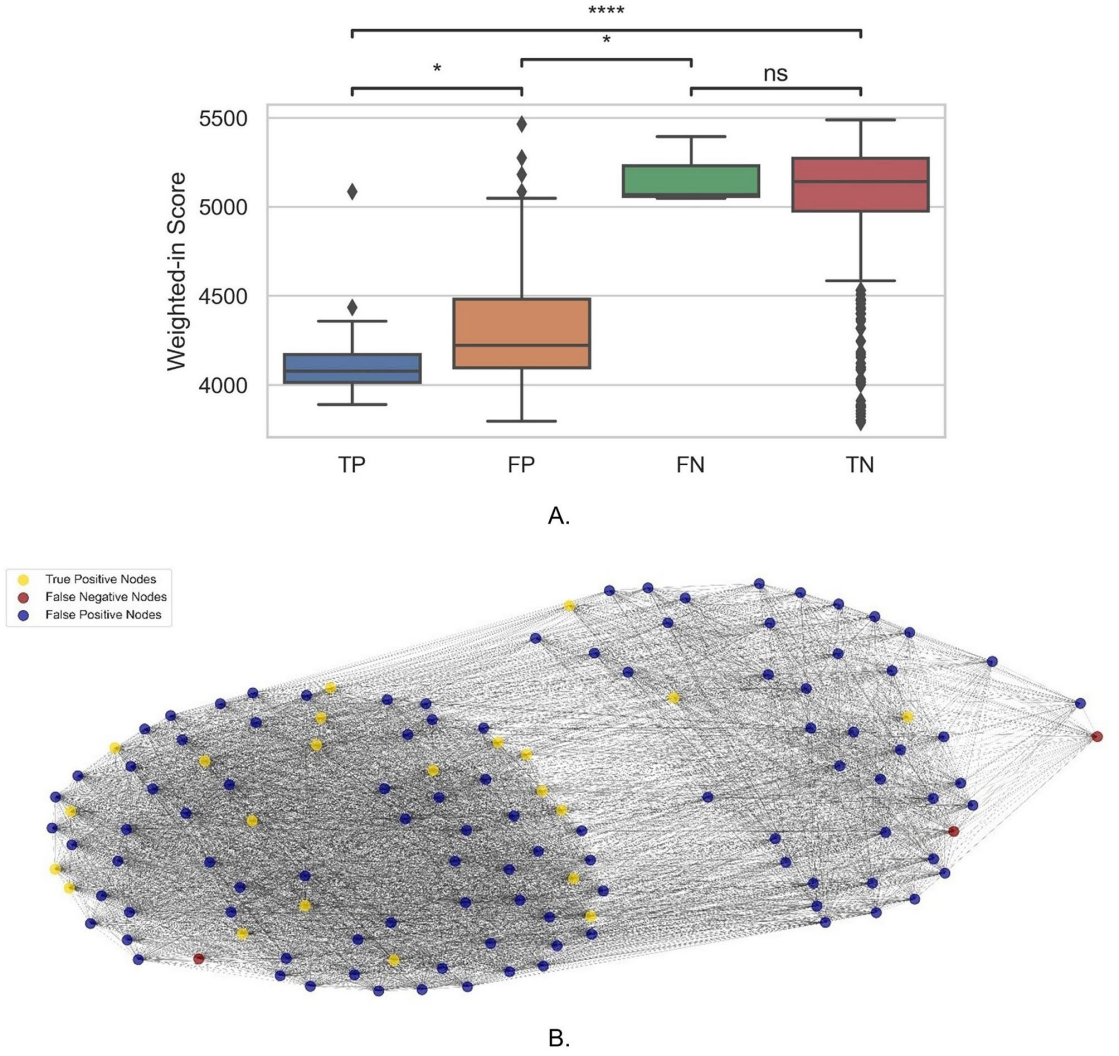
Weighted-in score with graph presentation of the testing data set. **(A)** Boxplot shows the weighted-in score for TP, FP, FN and TN cases. **(B)** Graph representation of the TP, FN and FP cases and their relationships (excluding the TN cases for clarity in presentation)

To gain a better understanding of the graph model, a subgroup analysis was performed on the 1116-patient test set (with 25 positive cases and 1091 negative cases). From the confusion matrix predicted by the GCN model, we divided the entire dataset into TP (*n* = 22), FP (*n* = 101), FN (*n* = 3) and TN (*n* = 1015) groups (for graphical representation, please refer to Fig. 4(B)). As shown in Supplementary Table 5, the majority of TP patients have a lower weighted-in score (4139.863 ± 256.268) than TN patients (5067.250 ± 333.273), with a significant difference in the Wilcoxon test (see Fig. 4(A)). However, the two erroneous predicted groups have the opposite characteristics, which may explain why the model predicted incorrectly for the 104 individuals (104 FP and 3 FN). Although predicted as positive, the FP patients’ weighed in score was significantly different from the TP group, while there was no significant difference between the FN and TN groups.

## Discussion

Predicting an individual‘s COVID-19 risk of mortality is critical for resource allocation and treatment optimization. This research proposes a novel use of GCNs to address severely imbalanced datasets. We applied this model to laboratory blood tests of COVID-19 patients in Hong Kong and compared it with CPH and traditional ML models. Traditional ML models perform classification on individual cases, ignoring their similarity or connection with other individuals. While GCNs leverage structural relationships within the data, enhancing predictive accuracy and robustness. By integrating demographic and laboratory data into a GCN framework, our approach addresses class imbalance and demonstrates significant improvements in prediction accuracy.

To address data imbalance, we also introduced oversampling algorithms for CPH and classical ML models. Our results show that traditional ML approaches are not sufficiently accurate for severely imbalanced datasets. By comparison with the second set of experiments, it is suggested that applying oversampling on a range of imbalanced datasets can help improve model performance when employing machine learning approaches. Finally, we have shown that the GCN model significantly outperformed all other ML methods and the baseline CPH models. Thus, adopting a population graph representation is a potential method that can significantly improve prediction when applied to this imbalanced dataset.

After that, we performed a subgroup analysis on the 1116-patient test set in order to gain a better understanding of the GCN prediction. We also generated a subgraph including the TP, FP and TN patients as Fig. 4(B) showed to help understand the structure of the population graph. The weight-in scores, which reflect the relationship between each node and its neighbours which revealed potential underlying reasons for the incorrect predictions.

The true positive group’s mean value was significantly less than the true negative group’s mean value, whereas the two false groups exhibited the opposite trend, revealing the source of the incorrect prediction. Interestingly, the significant differences between the TP and FP indicate that there is still room for model performance improvement. Additionally, the FN group does not differ significantly from the TN group highlighting the need for distinction between these cohorts, although the number of FN was low (*n* = 3). Finally, because the population graph’s edge weights were calculated using the patients’ age and gender information, we observe that our models were consistent with the contemporary literature, which indicates that age and gender are critical predictors of COVID-19 mortality, indicating that GCN prediction is meaningful from a medical standpoint.

Our research has several advantages. First, we used a large population-based dataset with a total number of 7606 cases across 42 hospitals. As a result, our data’s sample size supports the generalizability and robustness of our approach. Second, as far as we are aware, this is the first work to model COVID-19 population-based data as a graph and apply state-of-the-art graph neural networks for the task of mortality prediction. Furthermore, we developed a new method for treating laboratory blood test data and non-laboratory data separately, modelling them as nodes features and edge weights separately, which provides intuitions for future studies on how to use multi-modality medical data more meaningfully. Finally, we demonstrated that using graph models to represent medical data is a meaningful and effective method for the survival rate prediction task by analysing the graph structure information subgroup.

There were several limitations to our study. First, due to incomplete digitisation of patients’ records across multiple hospitals, we lack information on patients’ presenting history, and admission vital signs, which have been identified as important factors in COVID-19 related deaths [26]. Second, while the blood tests were provided, we lack individual medical treatment information after being confirmed as COVID-19 cases, which may impact an individual’s survival. Third, the current approach is utilised on a binary prediction task, and performance on multi-class prediction will need to be further investigated. Finally, we only used this graph model with the Hong Kong population, with relatively low case numbers and mortality compared to other regions. Whether this model can generalise to other regions need to be tested in the future.

## Conclusion

A series of experiments demonstrated that this population-based GCN out-performed all other comparators and demonstrated good discriminability between low- and high-risk individuals. This graph method initiatively showed a new direction to embed different kinds of clinical data (blood samples and patient basic information) for a very imbalanced dataset.

### Electronic supplementary material

Below is the link to the electronic supplementary material.

Supplementary Material 1

## Data Availability

Individual data points generated and/or analysed during the current study are not publicly available due to patient privacy concerns. Additional summarised data for the cohorts are available from the corresponding author on reasonable request.
