# Supplementary material for: Admission blood tests predicting survival of SARS-CoV-2 infected patients: a practical implementation of graph convolution network in imbalance dataset
Source: BMC Infect Dis. 2024 Aug 9;24:803. doi: 10.1186/s12879-024-09699-x (PMC11313168; doi:10.1186/s12879-024-09699-x)
Supplement: Supplementary file 1 — Supplementary Material 1 [file 12879_2024_9699_MOESM1_ESM.docx]

**Supplementary I: TRIPOD Checklist: Prediction Model Development and Validation**

| **Section/Topic** | **Item** |  | **Checklist Item** | **Page** |
| --- | --- | --- | --- | --- |
| **Title and abstract** | | | | |
| Title | 1 | D;V | Identify the study as developing and/or validating a multivariable prediction model, the target population, and the outcome to be predicted. | 1 |
| Abstract | 2 | D;V | Provide a summary of objectives, study design, setting, participants, sample size, predictors, outcome, statistical analysis, results, and conclusions. | 1 |
| **Introduction** | | | | |
| Background and objectives | 3a | D;V | Explain the medical context (including whether diagnostic or prognostic) and rationale for developing or validating the multivariable prediction model, including references to existing models. | 2 |
|  | 3b | D;V | Specify the objectives, including whether the study describes the development or validation of the model or both. | 2 |
| **Methods** | | | | |
| Source of data | 4a | D;V | Describe the study design or source of data (e.g., randomized trial, cohort, or registry data), separately for the development and validation data sets, if applicable. | 2 |
|  | 4b | D;V | Specify the key study dates, including start of accrual; end of accrual; and, if applicable, end of follow-up. | 2 |
| Participants | 5a | D;V | Specify key elements of the study setting (e.g., primary care, secondary care, general population) including number and location of centres. | 2 |
|  | 5b | D;V | Describe eligibility criteria for participants. | 2 |
|  | 5c | D;V | Give details of treatments received, if relevant. | NA |
| Outcome | 6a | D;V | Clearly define the outcome that is predicted by the prediction model, including how and when assessed. | 2 |
|  | 6b | D;V | Report any actions to blind assessment of the outcome to be predicted. | NA |
| Predictors | 7a | D;V | Clearly define all predictors used in developing the multivariable prediction model, including how and when they were measured. | 2 |
|  | 7b | D;V | Report any actions to blind assessment of predictors for the outcome and other predictors. | NA |
| Sample size | 8 | D;V | Explain how the study size was arrived at. | 2 |
| Missing data | 9 | D;V | Describe how missing data were handled (e.g., complete-case analysis, single imputation, multiple imputation) with details of any imputation method. | 2 |
| Statistical analysis methods | 10a | D | Describe how predictors were handled in the analyses. | 3-4 |
|  | 10b | D | Specify type of model, all model-building procedures (including any predictor selection), and method for internal validation. | 3-4 |
|  | 10c | V | For validation, describe how the predictions were calculated. | 3 |
|  | 10d | D;V | Specify all measures used to assess model performance and, if relevant, to compare multiple models. | 5 |
|  | 10e | V | Describe any model updating (e.g., recalibration) arising from the validation, if done. | 5 |
| Risk groups | 11 | D;V | Provide details on how risk groups were created, if done. | 4 |
| Development vs. validation | 12 | V | For validation, identify any differences from the development data in setting, eligibility criteria, outcome, and predictors. |  |
| **Results** | | | | |
| Participants | 13a | D;V | Describe the flow of participants through the study, including the number of participants with and without the outcome and, if applicable, a summary of the follow-up time. A diagram may be helpful. | 3 |
|  | 13b | D;V | Describe the characteristics of the participants (basic demographics, clinical features, available predictors), including the number of participants with missing data for predictors and outcome. | Table 1 |
|  | 13c | V | For validation, show a comparison with the development data of the distribution of important variables (demographics, predictors and outcome). | Supp Table 1 |
| Model development | 14a | D | Specify the number of participants and outcome events in each analysis. | 4 |
|  | 14b | D | If done, report the unadjusted association between each candidate predictor and outcome. | NA |
| Model specification | 15a | D | Present the full prediction model to allow predictions for individuals (i.e., all regression coefficients, and model intercept or baseline survival at a given time point). | 4 |
|  | 15b | D | Explain how to use the prediction model. | 4-5 |
| Model performance | 16 | D;V | Report performance measures (with CIs) for the prediction model. | Table 2 |
| Model-updating | 17 | V | If done, report the results from any model updating (i.e., model specification, model performance). | 4-5 |
| **Discussion** | | | | |
| Limitations | 18 | D;V | Discuss any limitations of the study (such as nonrepresentative sample, few events per predictor, missing data). | 5 |
| Interpretation | 19a | V | For validation, discuss the results with reference to performance in the development data, and any other validation data. | 4 |
|  | 19b | D;V | Give an overall interpretation of the results, considering objectives, limitations, results from similar studies, and other relevant evidence. | 4-5 |
| Implications | 20 | D;V | Discuss the potential clinical use of the model and implications for future research. | 5 |
| **Other information** | | | | |
| Supplementary information | 21 | D;V | Provide information about the availability of supplementary resources, such as study protocol, Web calculator, and data sets. | Attached separately |
| Funding | 22 | D;V | Give the source of funding and the role of the funders for the present study. | NA |

**Supplementary II: Data Preparation**

***Supplementary Figure 1:* Different laboratory parameters for COVID-19 patients in 2020, classified by status (broken black line = alive; solid red line = dead) and gender (male/female).**

***Supplementary Table 1*: Demographics and clinical characteristics of Training/validation and Testing Dataset**

|  |  | **Training and validation, n = 6465** | **Testing, n = 1141** | **Independent sample t-test** |
| --- | --- | --- | --- | --- |
| **Full Name** | **(Unit; Normal reference range)** | **Mean ± SD**  **(95% CI)** | | **p-value** |
| Age | Years | 46·98±19·14,  (46·51 - 47·45) | 46·70±19·17,  (45·59 - 47·81) | 0·6513 |
| Gender (Male) | Count; % | 3155  (48·80%) | 542  (47·50%) | NA |
| Haemoglobin | (g/dL; 11·7-14·9) | 13·57±1·66,  (13·53 - 13·61) | 13·56±1·61,  (13·46 - 13·65) | 0·8690 |
| Haematocrit | (L/L; 0·35-0·45) | 0·40±0·05,  (0·40 - 0·40) | 0·40±0·04,  (0·40 - 0·40) | 0·7663 |
| White Blood Cell count | (10^9^/L; 3·7-9·2) | 5·66±2·14,  (5·61 - 5·71) | 5·66±2·14,  (5·53 - 5·78) | 0·9569 |
| Neutrophil count | (10^9^/L; 1·7-5·8) | 3·58±1·86,  (3·53 - 3·62) | 3·57±1·79,  (3·47 - 3·67) | 0·9400 |
| Monocyte count | (10^9^/L; 0·1-0·8) | 0·54±0·24,  (0·54 - 0·55) | 0·55±0·24,  (0·53 - 0·56) | 0·5407 |
| Lymphocyte count | (10^9^/L; 1·0-3·1) | 1·44±0·75,  (1·42 - 1·46) | 1·46±0·98,  (1·40 - 1·51) | 0·4533 |
| Platelet | (10^9^/L; 145-370) | 225·02±76·00,  (223·17 - 226·87) | 222·09±5·26,  (217·72 - 226·46) | 0·2294 |
| Sodium | (mmol/L; 136-145) | 138·11±3·12,  (138·03 - 138·18) | 138·11±3·10,  (137·93 - 138·29) | 0·9374 |
| Potassium | (mmol/L; 3·4-4·8) | 3·82±0·44,  (3·81 - 3·83) | 3·78±0·43,  (3·76 - 3·81) | 0·0107 |
| Creatinine | (µmol/L; 49·0-90·0) | 75·05±47·27,  (73·90 - 76·21) | 74·42±38·08,  (72·21 - 76·63) | 0·6694 |
| Urea | (mmol/L; 2·8-8·1) | 4·32±2·29,  (4·26 - 4·38) | 4·24±2·34,  (4·10 - 4·37) | 0·2646 |
| Albumin | (g/L; 35·0-52·0) | 40·14±4·99,  (40·02 - 40·26) | 40·02±4·98,  (39·73 - 40·31) | 0·4505 |
| Alkaline phosphatase | (µ/L; 30-120) | 74·75±43·33,  (73·70 - 75·81) | 74·95±42·27,  (72·50 - 77·41) | 0·8844 |
| Total bilirubin | (µmol/L; 5·0-21·0) | 8·93±5·43,  (8·80 - 9·07) | 8·88±5·49,  (8·56 - 9·19) | 0·7424 |
| Alanine aminotransferase | (µ/L; 0·0-34·4) | 31·96±52·44,  (30·68 - 33·24) | 30·96±26·47,  (29·42 - 32·49) | 0·5278 |
| Lactate dehydrogenase | (µ/L; 0·0-246·4) | 213·20±82·64,  (211·18 - 215·21) | 216·14±84·81,  (211·22 - 221·06) | 0·2777 |
| Creatine kinase | (µ/L; 39-308) | 145·54±291·87,  (138·42 - 152·65) | 141·87±245·06,  (127·65 - 156·09) | 0·7068 |
| C-reactive protein | (mg/dL; 0·0-5·0) | 1·65±3·40,  (1·57 - 1·74) | 1·57±3·12,  (1·39 - 1·75) | 0·4835 |

**Supplementary III: Traditional ML methods**

Logistic Regression (LR) is one of the machine learning methods used to solve binary classification problems and is used to estimate the probability of a particular thing. However, the result of logistic regression is not a probability value in the mathematical definition and cannot be used directly as a probability value. Logistic Regression and Linear Regression are both generalized linear models. However, logistic regression assumes that the dependent variable y follows a Bernoulli distribution, while linear regression assumes that the dependent variable y follows a Gaussian distribution. Therefore, there are many similarities with linear regression. A logistic regression model has two hyper-parameters to tune, i.e. the penalty (e.g. L1-norm or L2-norm), the regularization strength *c* and the optimization solvers (e.g. Limited-memory BFGS (lbfgs) solver, liblinear solver and newton- conjugate gradient solver (newton-cg)).

One of the most common dimension reduction approaches is linear discriminant analysis (LDA). However, it is focused initially on a single-labelled issue. To quantify class separability, LDA uses between-class scatter and within-class scatter. Thus, it is an appropriate clustering structure for classification when the distance between classes is maximum, and the scatters within classes are low. A LDA model has one hyper-parameter to tune, i.e. the optimization solver, e.g. singular value decomposition (svd), least squares solution (lsqr) and eigenvalue decomposition (eigen).

The K-Nearest Neighbours (KNN) method, which bases on the Supervised Learning approach, is one of the simplest and easiest-to-implement Machine Learning algorithms. It can tackle both classification and regression problems. The KNN algorithm assumes that the new case/data and existing cases are comparable and places the new case in the most similar category to existing ones. Thus, the KNN algorithm saves all available data and classifies a new data point based on its similarity to the existing data. The KNN algorithm can easily classify new data into an appropriate suite category as it emerges. A KNN model has three hyper-parameters to tune, i.e. the weight function (e.g. uniform weights and distance weight), distance metric (e.g. Euclidean distance, Manhattan distance and Minkowski distance), and the number of neighbours to use (n_neighbor).

The Gaussian process is a machine learning method developed based on statistical learning theory and Bayesian theory. It is suitable for dealing with complex regression problems such as high dimensions, small samples, and nonlinearity and has strong generalization ability. It directly models the function to generate a non-parametric model. One of the outstanding advantages of this is that it can simulate any black-box function and simulate uncertainty. This quantification of uncertainty is essential. For example, when we can request more data, relying on the Gaussian process, we can explore the most unlikely data areas for efficient training. Moreover, it is compatible with neural networks and support vector machines. In comparison, Gaussian Process has the advantages of easy implementation, adaptive acquisition of hyper-parameters, flexible non-parametric inference, and probabilistic output. A Gaussian process model does not have hyper-parameter to tune.

A support vector machine (SVM) is a supervised machine learning model that employs classification techniques for two-group classification issues. SVM models can categorize deceased patients after being given sets of labelled training data for each category. A support vector machine has two hyper-parameters to tune, i.e. the kernel function (e.g. polynomial kernel (poly), the radial basis function kernel (rbf)) and the sigmoid kernel (sigmoid)) and a regularization parameter *c*.

A XGBoost is an ensemble machine learning algorithm which is also known as gradient boosting framework. It utilizes decision trees as base learners and employs regularization techniques to enhance model generalization. XGBoost is famous for its computational efficiency, offering efficient processing, insightful feature importance analysis, and seamless handling of missing values. A XGBoost model has three hyper-parameters to tune, i.e. the maximum tree depth for the base learners (max_depth), learning rate (eta), and the number of gradient boosted trees (n_estimators)

In supplementary Table 2, we summarised our experiment results for choosing the optimal hyper-parameters for each machine learning model used in this study. We measured the area under the ROC curve to validate the performance of models with various hyper-parameter settings. To summarise, for the logistic regression, we used the liblinear solver with regularisation c = 100. For the LDA model, the three solvers yielded the same AUC. Since svd gave the minimum calculation time, we chose svd. For KNN model, the model with Manhattan metric, distance as the weights and 19 neighbours gave the best AUC. For the SVM with rbf kernel and *c*=50 gave the best AUC. For XGBoost method, when eta=0.3, maximum depth = 3 and used 200 estimators, the model yielded the best AUC.

It is rare to encounter a situation where the positive and negative sample data ratio is equal in clinical data analysis. Usually, the vast majority are positive samples, and there are only a few (a few or a dozen) negative samples. In our case, whether it is LDA, KNN, Gaussian, or SVM, the effect of directly using the data set for learning will not be very good because the learning results of these methods will be biased towards the one with more samples. On the other hand, when evaluating the learning results, if the negative samples account for 98.2% (7464/7606) and the positive samples only account for 1·8% (142/7606), we do not even need to learn and directly predict all-new samples as survival case, the accuracy rate can reach 98·2%, and the recall rate is meager. Therefore, it is necessary to deal with unbalanced data before learning a model.

Therefore, the SMOTE (Synthetic Minority Oversampling Technique), synthetic minority oversampling technique was introduced. It is an improved scheme based on the random oversampling algorithm. However, because random oversampling adopts a simple copy sample strategy to increase the minority samples, it is easy to cause the problem of model overfitting. That is, the information learned by the model is too unique and not enough Generalization. Therefore, the basic idea of the SMOTE algorithm is to analyze the minority samples and artificially synthesize new samples based on the minority samples and add them to the data set. (please refer to supplementary Figure 3)

Specifically, the distribution of the hostile class sample determines its selectable neighbors. If a negative class sample is at the edge of the distribution of the hostile class sample set, the "artificial" samples generated by the negative class sample and neighboring samples will also be at this edge. As a result, it will become more marginalized, which blurs the boundary between positive and negative samples, and makes the boundary more and more blurred.

The limitations of the SMOTE algorithm There are two main problems with this algorithm First, there is a certain degree of blindness when selecting the nearest neighbors. It can be seen from the above algorithm flow that in the process of algorithm execution, the K value needs to be determined, that is, how many neighbor samples to choose, which needs to be solved by the user. It can be seen from the definition of K value that the lower limit of K value is M value (M value is the number of neighbor samples randomly selected from K neighbors, and there is M < K), and the size of M can be based on negative samples The number, the number of positive samples and the final balance rate of the data set are determined. However, there is no way to determine the upper limit of the K value, and it can only be tested repeatedly according to specific data sets. Therefore, how to determine the value of K to make the algorithm reach the best is unknown.

Supplementary Figure 2: Comparing SMOTE, random over- and under sampling methods using five-fold cross-validation.


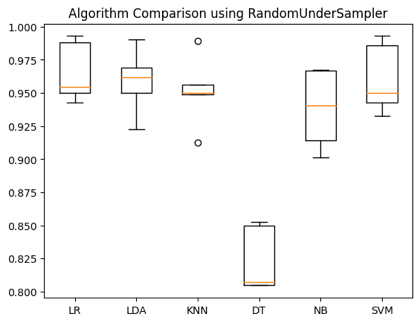

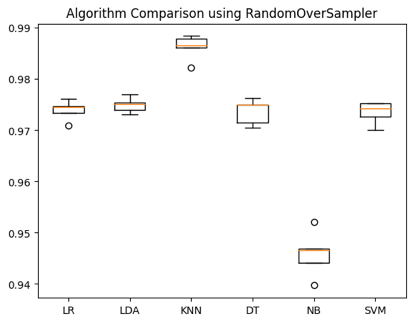

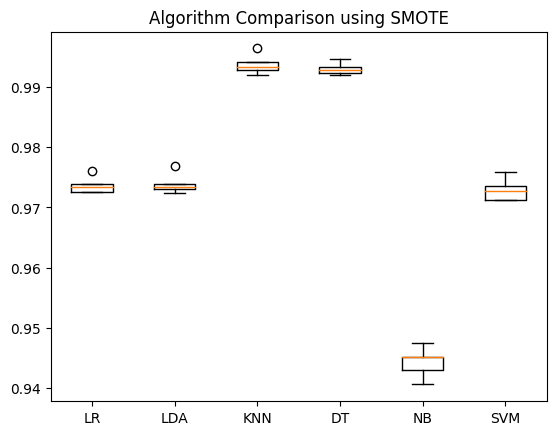


Supplementary Table 2. The classification results of for various machine learning models with different hyper-parameters.

| ML method | Hyper-parameters | | Optimal hyper-parameters on validation set | | |
| --- | --- | --- | --- | --- | --- |
|  |  |  | Rank | Hyper parameters | AUC |
| O-Logistic Regression | Optimisation solvers | (1) Newton-cg  (2) liblinear  (3) lbfgs | 1st | c = 100  Solver = liblinear | 0.97453 |
|  | regularisation c-value | 0.01, 0.1, 1.0, 10, 100 | 2nd | C = 100  Solver = newton-cg | 0.97452 |
|  | - | | 3rd | C=100  Solver = lbfgs | 0.97451 |
| O-Linear Discriminant Analysis | Optimisation solver | (1) svd  (2) lsqr  (3) eigen | 1st | svd | 0.97459 |
|  |  |  | 2nd | Lsqr | 0.97459 |
|  |  |  | 3rd | eigen | 0.97459 |
| O-K-Nearest Neighbor | weights | (1) uniform  (2) distance | 1st | Metric = manhattan  n-neighbors = 19  Weights = distance | 0.99196 |
|  | metric | (1) Euclidean  (2) Manhattan  (3) Minkowski | 2nd | Metric = manhattan  n-neighbors = 19  Weights = uniform | 0.99189 |
|  | n-neighbors | 1,3,5,7,9,11,13,  15,17,19,21 | 3rd | Metric = manhattan  n-neighbors = 17  Weights = distance | 0.99155 |
| O-Support vector machine | kernel | poly, rbf, sigmoid | 1st | C = 50  kernel = rbf | 0.98904 |
|  | regularisation C | 50, 10, 1.0, 0.1, 0.01 | 2nd | C = 50  kernel = poly | 0.98677 |
|  | - | | 3rd | C = 10  kernel = rbf | 0.98441 |
| O-XGBoost | Max depth | 1,2,3 | 1st | eta = 0.3  max_depth 3 n_estimators = 200 | 0.99752 |
|  | eta | 0.01, 0.1, 0.3 | 2nd | eta = 0.3  max_depth 3  n_estimators = 100 | 0.99675 |
|  | Number of estimators | 50,100,200 | 3rd | eta = 0.1  max_depth 3 n_estimators = 200 | 0.99657 |

**
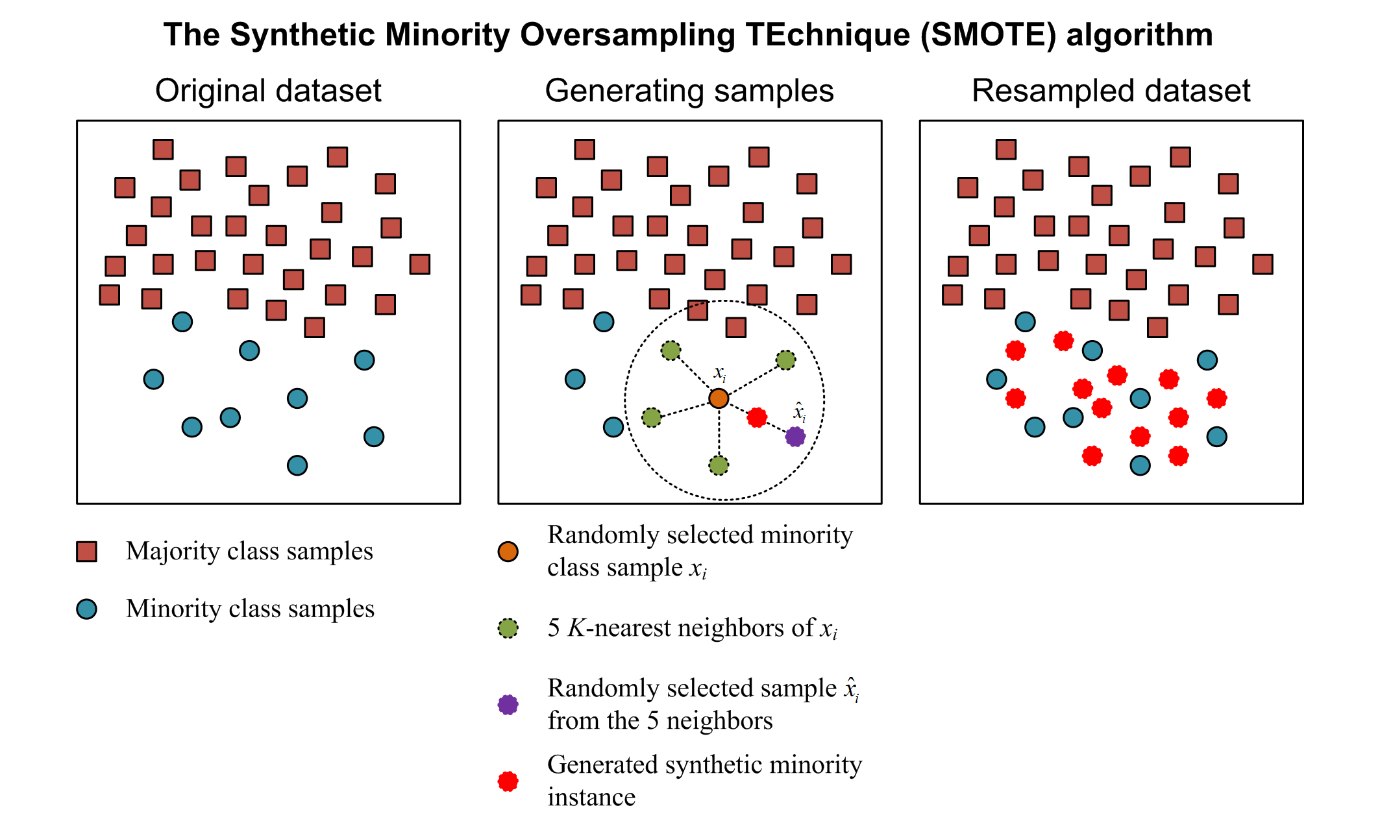
*Supplementary Figure 3*: Overview of the SMOTE algorithm**

**Supplementary IV: Similarity Score Definition**

Considering two patients’ nodes *n_i_*, *n_j_* and their sex, age information *s_i_*, *s_j_* and *a_i_*, *a_j_* , we defined a Similarity score function $SS$ to calculate the edge weight between the two nodes

In this case, the value of *SS*_(_*n_i_*, *n_j_*_)_ could be 0, 1 or 2. If *SS*_(_*n_i_*, *n_j_*_)_ was larger than 0, there will be an edge between nodes *n_i_* and *n_j_* . and the larger value of *SS*_(_*n_i_*, *n_j_*_)_ means that nodes *n_i_* and *n_j_* were stronger connected in the population graph since they shared more common properties from the aspect of non-clinical data.

**Supplementary V: Delong's test results**

***Supplementary Table 3***: **p-values of Delong's test comparing the difference between ROC curves of 7 different models.**

| **p-value** | **O-CPH** | **O-LR** | **O-LDA** | **O-KNN** | **O-GAUSSIAN** | **O-SVM** | **GCN** |
| --- | --- | --- | --- | --- | --- | --- | --- |
| **O-CPH** | NA | ≤0·001*** | ≤0·001*** | ≤0·001*** | ≤0·001*** | ≤0·001*** | ≤0·001*** |
| **O-LR** |  | NA | ns | ns | ≤0·05* | ns | ≤0·01** |
| **O-LDA** |  |  | NA | ns | ≤0·05* | ns | ≤0·001*** |
| **O-KMEAN** |  |  |  | NA | ns | ns | ≤0·01** |
| **O-GAUSSIAN** |  |  |  |  | NA | ns | ≤0·001*** |
| **O-SVM** |  |  |  |  |  | NA | ≤0·01** |
| **GCN** |  |  |  |  |  |  | NA |

ns: p > 0·05, *: p≤ 0·05, **p ≤0·01, *** p≤0·001

***Supplementary Table 4***: **Five-folder cross validation results comparing GCN with oversampled ML models. During the cross-validation process, each train/validation set were oversampled while the test set was not oversampled.**

|  | **ML models** | **AUC**  **Mean** | **AUC**  **std** |
| --- | --- | --- | --- |
| **Traditional Models based on oversampled data** | **O-LR** | 0·897 | 0.022 |
|  | **O-LDA** | 0·898 | 0.019 |
|  | **O-KNN** | 0·886 | 0.038 |
|  | **O-GAUSSIAN** | 0·801 | 0.030 |
|  | **O-SVM** | 0.731 | 0.052 |
|  | **O-XGB** | 0.694 | 0.022 |
| **Our proposed method** | **GCN** | 0·953 | 0.031 |

**Supplementary VI: Weighted-in score results**

***Supplementary Table 5*: Weighted-in score for True positive, false positive, false negative and true negative cases.**

|  | **True Positive** | **False Positive** | **False Negative** | **True Negative** |
| --- | --- | --- | --- | --- |
| **Number of Nodes** | 22 | 101 | 3 | 1015 |
| **Mean** $\boldsymbol{\pm}$ **SD of weighted-in score** | 4139·863$\pm$256·268 | 4332·049$\pm$350·936 | 5170·666$\pm$194·561 | 5067·250$\pm$333·273 |

**Supplementary VII: KM Curve**

**
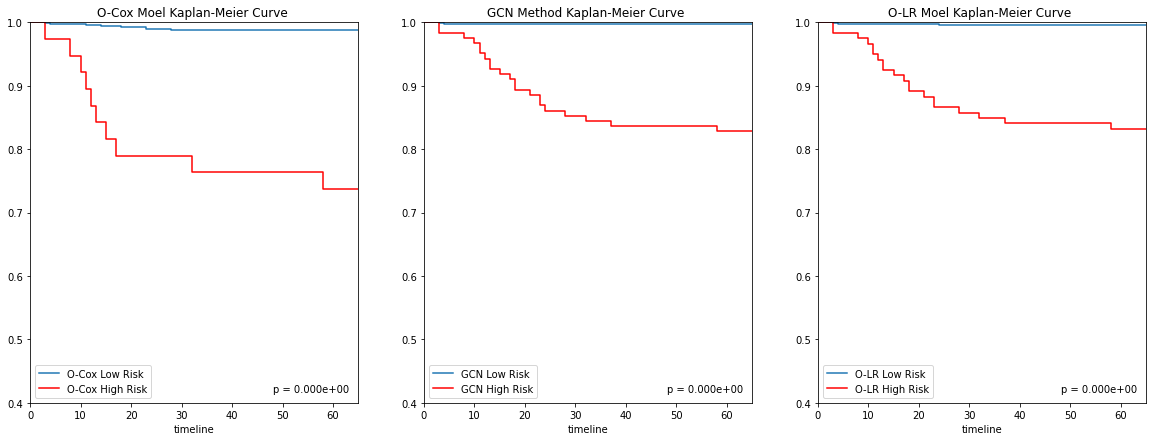
**

**Supplementary Figure 4: KM Curve for O-Cox, GCN and O-LR models**
